# Unleashing the importance of creativity, experience and intellectual capital in the adaptation of export marketing strategy and competitive position

**DOI:** 10.1371/journal.pone.0241670

**Published:** 2020-11-03

**Authors:** Salman Ali, Guihua Li, Yousaf Latif

**Affiliations:** 1 Business School & Binhai College of Nankai University, Tianjin, PR, China; 2 School of Economics, Nankai University, Tianjin, PR, China; University of Almeria, SPAIN

## Abstract

Export marketing strategy has become an exciting research topic in strategic management literature because of its momentous role in sustainable competitive advantage and performance of firms. However, it is not yet recognized what factors enable top management team in adaptation of the export marketing strategy. This research aims to unleash how the intangible skills; creativity, business experience and intellectual capital facilitate marketing managers in adaptation of the expert marketing strategy (product, price, promotion and distribution) that can spur sustainable competitive performance. We collected data from 293 SMEs and used structural equation modeling for testing the hypotheses. The results indicate that the intangible skills; creativity, experience and intellectual capital do not directly contribute to sustainable competitive performance. However, creativity has a significant influence on product, price, promotion and distribution strategy, experience has a significant influence on product, price and promotion strategy and intellectual capital is only a significant predictor of product strategy. In the dimensions of export marketing strategy, product, price and distribution significantly while promotion does not significantly contribute to sustainable competitive performance. Moreover, export marketing strategy adaptation fully mediates the relationship between creativity and sustainable competitive performance as well as between experience and sustainable competitive performance while it does not mediate the path between intellectual capital and sustainable competitive performance. The findings recommend SMEs to emphasize highly skilled marketing staff who have competencies (experience, creative and intellectual) in order to build an effective export marketing strategy—resulting sustainable competitive performance. Further implications are discussed.

## 1. Introduction

The concept of an export marketing strategy has been recognized for more than fifty years by scholars and practitioners in the field of international business and international marketing [1, 2]. The term became very popular in academia and research because of its significant role in sale growth and superior profitability [3, 4]. Export marketing strategy is defined as the strategic response of the interplay of the internal and external forces faced by a firm in a foreign market [5]. In a marketer carefully investigates his/her strategy and adjusts it with foreign market surroundings, there is great possibility of high performance [3]. It ultimately illustrates that a sound export marketing strategy enables marketers in succeeding their products in competitive markets which in turn provide high performance. In the current changing market needs, the ability to generate and market creative ideas regarding marketing programs and new products is key to success of an organization [6]. Additionally, it is argued that due to the globalization, competition and rapid changes in technology, the success of many organizations has become progressively more dependent on binning innovative products to market. However, innovative depends on new ideas, creativity and skills [7]. Unfortunately, many organizations eventually fail in launching a suitable product for customers because of ineffective marketing strategy and poor skills [8, 9]. Managers should consider creative marketing strategies and creative skills during implementation of marketing strategy in order to survive in the dynamic market [10]. The literature has highlighted the importance of different marketing assets in business success and competitiveness, it is still far from reaching a consolidated positon on the topic how intangible skills enables marketing managers in adaptation a suitable export marketing strategy that results high profitability. In other words, it is not yet known what kinds of skills and abilities enable marketers in building and adopting export marketing strategy (in terms of product, price, promotion and distribution) that might useful for sustainable competitive performance.

There are following reasons why the particular intangible skills of marketing managers are vital for export marketing strategy adaptation. First, we followed the suggestions of Ying, Hassan [11] who tested the role of the intangible skills; financial literacy, business experience and intellectual capital of financial managers and owners in achieving sustainable competitive advantage and suggested the factors as more vital for innovative and performance. However, rare or perhaps no attention is given to intangible skills in export marketing strategy. Second, out of several problems, tangible resources constraints is the key issue that hinders innovativeness, new marketing enter, new product development and growth of SMEs [12, 13]. Hence, they prefer to gain advantage of intangible resources and capabilities [12, 14]. Third, we use creativity as it is more concrete variable and generally viewed as a crucial predictor of innovation [15]. For instance, Amabile [16] describe: "All innovation begins with creative ideas”. It is very important for product development team because it influences product development activities when main goal is to bring innovation [6]. We use experience that encompasses both; experience in domestic firms and trading and in international business (import/export). We perceive that the experience might be decisive in adopting export marketing strategy that resulting an increased sales. Reasons behind using intellectual capital are analytical skills and ideas enabling marketers in launching new products and new processes. For instance, Sardo and Serrasqueiro [17] claimed that intellectual managers are proactive in seizing new opportunities in the market, thereby resulting in a sustainable position. Heirati and O’Cass [18] claimed that most of the products are failed in international markets in the initial stage due to ignoring or giving less attention to customers choices, demands and preference and traditional approaches use by marketing managers [19]. We perceive that the intangible skills enable marketing managers to deeply investigate the customers’ choices and international market trend, thus bringing suitable products to the markets.

This research contributes to the Resource Based View (RBV) theory—initially proposed by [20]. This theory sheds light on the prominence of both tangible resources (technology, finance, new products, and infrastructure etc.) and intangible resources (intellectual capital, experience, capabilities, skills, networking and strategy etc.) which facilitate organizations in getting sustainable competitive performance [11, 12, 21]. The theory has widely discussed in management, economics and business literature in both theoretical and empirical contexts [11, 14, 22]. However, the particular intangible skills; creativity, experience and intellectual capital from marketing managers perspective in export marketing strategy adaptation and sustainable performance have been neglected. The present study advances the understanding of RBV theory and suggests useful implications for marketing managers and policy makers. The present study facilitates marketing managers of SMEs in understanding the relative importance of the particular intangible resources for export marketing strategy adaptation. Moreover, it enables Chinese and Pakistani SMEs to formulate their strategies for CPEC and international trade. In general the present study helps marketing managers in reconfiguring their strategies for product, price, promotion and distribution for national and internal product launching.

**Fig 1:**
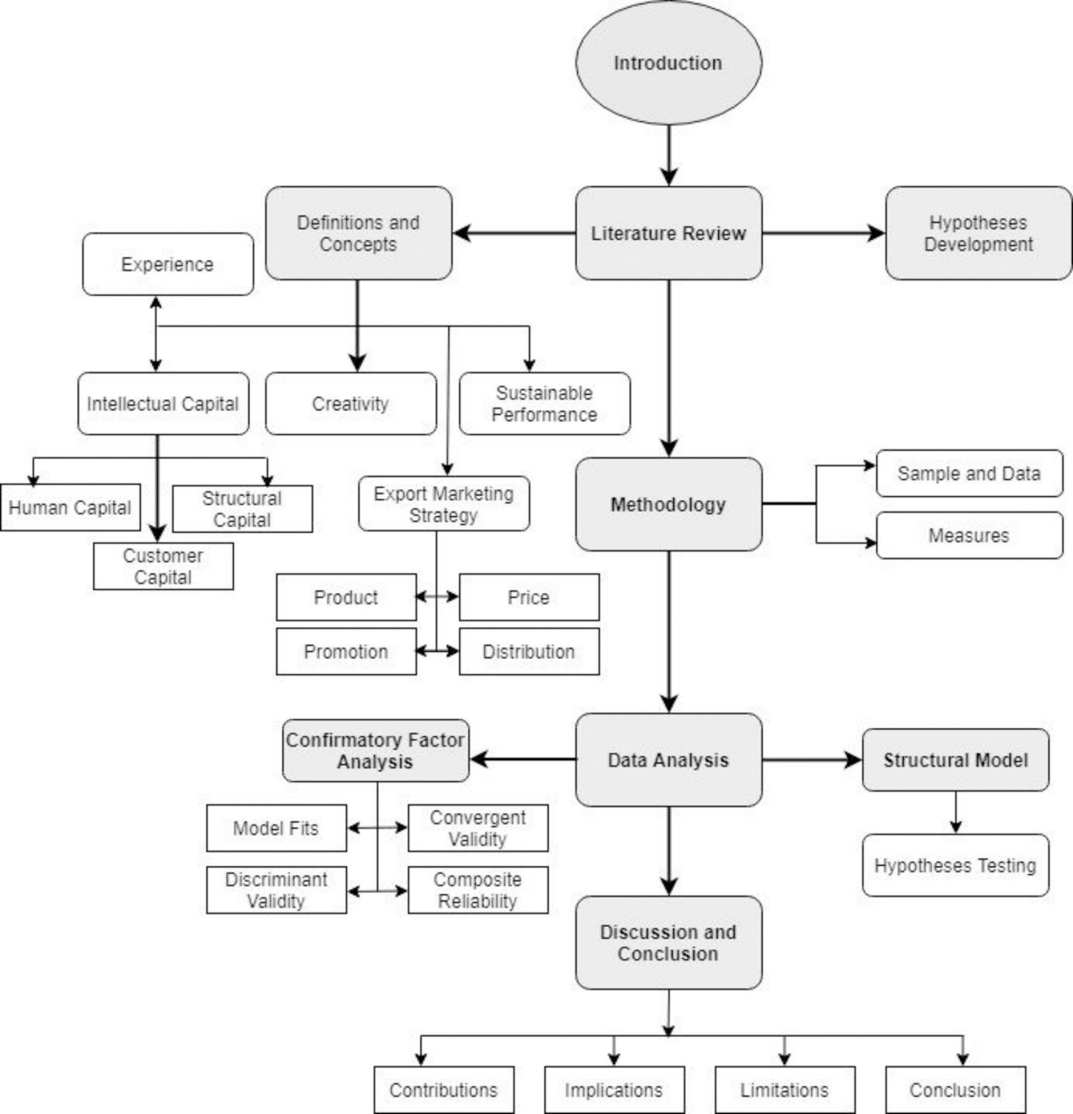
Organization of the study.

Fig 1 illustrates organization of the study. As shows in the figure, the first part start with introduction, then review of relevant literature and then we have discussed methodology. In the fourth section, we discussed data analysis and then finally we discussed recommendations and conclusion.

## 2. Theoretical background and hypotheses development

### 2.1 Creativity

In the field of marketing, creativity is conceptualized as the organizational level to enlighten the extent to which a firm offers valuable and unique solutions to market problems and issues [23–25]. Agnihotri, Rapp [23] developed a model to demonstrate that creative boundary spanners enable enterprises to spur their service delivery to enrich their performance. Marketing literature has argued two major elements of creativity; novelty and usefulness [26].

Amabile [27] further shed light on creativity and creative culture and discussed creativity as “a novel, useful, correct, appropriate and value response to the task at the hand and the task is heuristic rather than algorithmic”. However, the creative process is the interrelationship of person, task and organization [28].

### 2.2 Experience

In business literature, experience demonstrates the ability and knowledge of sale, purchase, supply chain, import and export etc. [29]. A manager or owner of business is considered experienced manager if he has worked in business industry for a short or long time. In the present study, we deem marketing managers as experienced managers if they have spent time in national or international trading, purchase and sale and adverting etc.

Intellectual capital is considssered as an intangible asset of organization that is used for gaining advantages such as sustainable competitive advantage and profitability [12]. Bontis, Bart [30] suggested three dimensions of intellectual capital: *Human capital* refers to all the skills, knowledge and capabilities accumulated by the employees working in an organization, *Structural capital* indicates overall system and programs used by an organization to solve problems and threats and to create values, *Relational capital* refers to the relationship and ties build by a company with customers, partners and supplier to gain benefits such as resources, information and help [31].

### 2.3 Export marketing strategy

Marketing strategy refers to policies and goals for prospective customers and turning them into real customers related to product, price, promotion and placement—thereby resulting sustainable competitive advantage. Export marketing strategy is related to using or following foreign marketing strategies for existing or new customers to enhance sale and profitability. In other concepts, export marketing strategy is defined as the strategic response of the interplay of the internal and external forces faced by a firm in considering foreign market [5]. In a marketer carefully investigates his/her strategy and adjusts it with foreign market surroundings, there is great possibility of high performance Šeinauskienė, Virvilaitė [3], (2019). In the present study, we define export marketing strategy as a plan and policy for improving products sales in the domestic and foreign markets in terms of price, promotion and distribution.

### 2.4 Sustainable competitive performance

It illustrates the superior performance; profit, assets, outputs and positon of an organization over others because of unique, rare and valuable resources [20, 32]. Considering the RBV theory [33] an enterprise secure a sustainable competitive positon in the market with adequate, rare, immutable and secured tangible (finance, technology, land and infrastructure) and intangible (intellectual capital, entrepreneurial ability, reputation, knowledge and information etc.) resources.

Considering the RBV theory [33] we argue that creativity, experience and intellectual capital of marketing managers are the key intangible resources—enabling them in the adaption of the best marketing strategy that results superior performance. For instance, claimed that many corporations flourish in announcing new products and new processes to the markets through advance marketing strategy that is based on creativity, skills and market orientation of the staff (e.g. marketing and products). These companies also gain sustainable competitive position in the market because of uniqueness and creativeness [20, 34]. Sraha, Raman Sharma [35] shed light on experience and knowledge of international business that facilitate managers in securing a stable positon and gaining high profit. Additionally, Khan, Yang [12] scrutinized that the intangible skill; intellectual capital is the key elements of securing sustainable competitive advantage and superior performance over competitors in emerging markets. Therefore, it is doubtless to say that certain intangible assets; creativity, experience and intellectual capital will empower marketing skills of marketers in terms of products, price, promotion and distribution that can configure performance of the enterprises.

### 2.5 Marketing creativity, export marketing strategy and SCP

Businesses with the most innovative and creative abilities have an opportunity horizon that facilitate them to restructure their process in a way which creative sustainable competitive advantage [10]. Creative marketing strategy facilitates new pricing models, expanded ways, customer driven supply networks, means for touching and innovative value propositions that respond to their specific interests and preferences. These marketing mixed elements and creative ideas enable firms to make unique abilities that cannot be copied by the competitors—thereby resulting sustainable competitive performance [10, 20].

In marketing studies, creatively is extensively used as programs, processes and actions that are significantly novel as compared to existing practices and actions [36, 37]. From individual perspective, creativity refers to activities and actions of individuals’ employees within an organization to constitute their capability to build something, which is novel and evocative within their work environment. However, from organizational perspective, creativity refers to actions, programs, tools, approaches and practices that constitute new behaviors with the organization. Although not empirically tested, individuals’ creativity enables organizations to adopt innovative practices and novel processes [38, 39]. Marketing strategy encompass newness in product, differentiation in price, process and promotion that is influenced by marketing managers creativity. For instance, Amabile [40] claimed that innovation in firms is significantly affected by employees’ creativity and skills. In other words, creative related skills enables organizations in attaining their objectives that are to improve innovative performance newness [41]. Creativity has been considered one of the significant factors of firms’ innovation and competitive performance when business face a big challenge in turbulent market [42].

Creativity exhibits facilitates organizations in solving their problems and provides a strong ground for building new products and process for their target markets and target customers [43]. An enterprise with creative skills has superior ability of producing new products and processes that are more likely unique, occupy a lucrative market position and cannot be replicated by their competitors [44, 45]. Zhao and Chadwick [46] claimed that majority organizations succeed in introducing new products and new processes to the markets through advance marketing strategy that is based on creativity, skills and market orientation of employees. These firms also gain sustainable competitive position in the market (difficult to seize the same position by their competitors) because of uniqueness and creativeness [20, 34]. With creative skills and abilities, an organization is able to develop and commercialize new products and process that are rare, unique, valuable and imperfectly imitable. These advantages (new product and new process that are gained through creativity) in turn facilitates firms in getting superior market performance and increased success [47]. Creativity significantly influences the internal process (strategy, competitive advantage and ideas) of a firms that in turn affect profitability and performance [42].

### 2.6 Business experience, export marketing strategy and SCP

Managers with international experience have relatively high intensity of international market knowledge, international market conditions and trends, customers, regulations, product processes and advertising [48]. Such experience enables owners and managers of enterprises to adopt the foreign marketing strategies and make a successful entry in international markets [49].

Experience (in foreign companies and trading) facilitates managers and owners of businesses in adopting international marketing strategy and international trading process—resulting superior market performance [50]. Broad experience of business activities plays a crucial role in marketing field because having information of customers’ knowledge, choices and intention help marketers in formulating marketing strategy accordingly [51].

Relevant experience enables product managers in accumulation of relevant information resources for uniqueness and newness of products. Hence, managers with experience are able to perform new product development tasks in an effective way [52].

Experienced marketing managers might have information market trends, customer demands, international products and international regulations which can help them in building an effective product and process that provide desirable outcomes [53]. Managers’ background and especially experience significantly influence marketing strategies and the way they form their advertising and marketing policies [54]. Managers with broad experience in marketing easily evaluate the markets, customers’ demands and choices. Hence, they have a low level of financial and physical loss and have a satisfactory level of profitability and performance [55]. Marketing managers with soft skills such as global experience, capability and understanding build effective export marketing strategy which in turn enhance their profitability and performance [56]. Managers who are connected with customers have understanding and knowledge of their customers’ anxieties and choices. Hence, they able to build the most suitable marketing strategy while disconnected managers underestimate their customers—resulting poor marketing strategy and undesirable performance [57].

As compare to less or no experience managers, high experiential managers proactively exploit new opportunities in markets and configure their strategies accordingly—thereby resulting satisfactory performance [21].

When managers have international experience and know the international trade tactics, they can easily commit export activities which result high performance and greater profitability [35]. Adequate knowledge and experience are very crucial in building innovative products and marketing strategy. It empowers managers in producing tolerably match products for customers and markets that result superior performance [58]. If product team (e.g. marketing department) has adequate knowledge, information and prior experience of pricing strategies of similar products, they will able to produce demandable and environmentally fit products [59].

### 2.7 Intellectual capital, export marketing strategy adaption and SCP

Intellectual capital is the ability of managers/owners which helps them in improving marketing assets and marketing resources that are important for high performance [22]. Intellectual capital is one of the important internal environmental factors that influences management strategy related to innovative and [60]. Newness, uniqueness and radically different products can be possible through intellectual skills and intellectual abilities [61]. IC configures internal processes of organizations-resulting newness in building new product strategy [62]. Marketing managers need new ideas, sufficient market knowledge and information when they build strategy for new product. In this case, IC enables them in getting heterogeneity knowledge [63]. High competitive organizations take advantage of getting new knowledge through IC when they create strategy for innovative and new product development [64].

SMEs are likely to invest in intangible resources (e.g. IC) as it saves their costs related to material, operation and advertising and helps in building unique and differentiated products that spur their sustainable competitive performance [12]. IC reconfigures the internal structure (product, process, operation and adverting) of organizations in a positive way that can significantly improves profitability [65].

Capabilities and skills are necessary in managers to effectively engage in export activities and export strategies. This in turn provide desirable outcomes and sustainable positon in the market [66].

Intellectual capital has become a popular determinant of global marketing strategy in business industry because it enables enterprises in achieving competitive advantage through building worthy global marketing strategy [67].

Marketing managers should have understanding and ability of understanding all the aspects that influence export marketing strategy, rather believing in narrow factors and only cultural issues. It can be possible when a firm has intellectual marketing managers who can judge the market deeply in order to configure profitability [68].

Innovativeness (product, process, marketing and organizational) and growth of an organization is dependent on its intellectual capital. It permits enterprises to grow in the market through structuring innovative strategies [60]. In the current era of business competition, enterprises need innovative strategies and innovative practices. One of the possible factors to adopt and generate innovation is intellectual capital [69]. Similarly, Buenechea‐Elberdin, Kianto [70] also scrutinized that the product and managerial innovation of a company are influenced by degree of intellectual capital. In turn, the innovative tactics contributes to performance. The indirect influence of intellectual capital on enterprises performance and profitability through innovative quality and innovative speed is also reported by Wang, Cai [71].

### 2.8 Export marketing strategy adaption and SCP

In the competitive atmosphere, businesses need to opt foreign marketing strategies in terms of product, price and promotion because it increases sale growth and profitability due to a high acceptance of the product in markets [72]. Adaptation of export marketing strategy is key to export performance and helps ventures in getting sustainable position in the market [4]. Drawing on the RBV theory, export marketing strategy researchers that marketing responsiveness and abilities of building new and innovative products enable enterprises in making sustainable position in the domestic and foreign markets [73, 74]. However, the RBV theory is criticized for being giving poor information of how resources and capabilities are developed by a firms in order to gain superior profit and a sustainable positon over other firms [75]. This limitation of the RBV theory is explained by dynamic capabilities theory and revealed that possession of resources and capabilities enable enterprises to earn sustainable market positon when they are fit to the environment [76, 77]. Considering the theme of dynamic capability theory, export marketing strategy entails complex skills and capabilities of managers that are enriched internal posture—thereby spurring profitability and positon [74]. Marketing strategies and capabilities are very important for high performance in competitive markets. Hence, marketing managers are required to adopt and favor unique marketing strategies [78]. An organization with intention to compete, earn profit and gain a sustainable positon in the market needs adjustment in the marketing strategy (especially adopting export marketing [74]). To enrich the notion, several researchers have shed light on the importance of adopting suitable marketing strategy that is necessary for superior performance. For instance, Menguc and Auh [79] claimed that organizations must consider product innovation strategy in their marketing strategies to ensure their superior performance and competitive position. Gregory, Ngo [80] shed light on export marketing strategy (distribution and communication) in the contexts of the RBV theory and described that the unique export marketing capability is crucial for high profit.

### 2.9 Hypotheses

H1. Creativity significantly contributes to the sustainable competitive performance.
H2. Business experience significantly contributes to the sustainable competitive performance.
H3. Intellectual Capital significantly contributes to the sustainable competitive performance.
H4. Creativity significantly facilitates marketing managers in export marketing strategy adaption (product, price, promotion and distribution).
H5. Business experience significantly facilitates marketing managers in export marketing strategy adaption (product, price, promotion and distribution).
H6. Intellectual Capital significantly facilitates marketing managers in export marketing strategy adaption (product, price, promotion and distribution).
H7. Export marketing strategy adaption (product, price, promotion and distribution) has a significant influence on sustainable competitive performance.
H8. Export marketing strategy adaption mediates the relationship between creativity and sustainable competitive performance.
H9. Export marketing strategy adaption mediates the relationship between experience and sustainable competitive performance.
H10. Export marketing strategy adaption mediates the relationship between intellectual capital and sustainable competitive performance.

**Fig 2:**
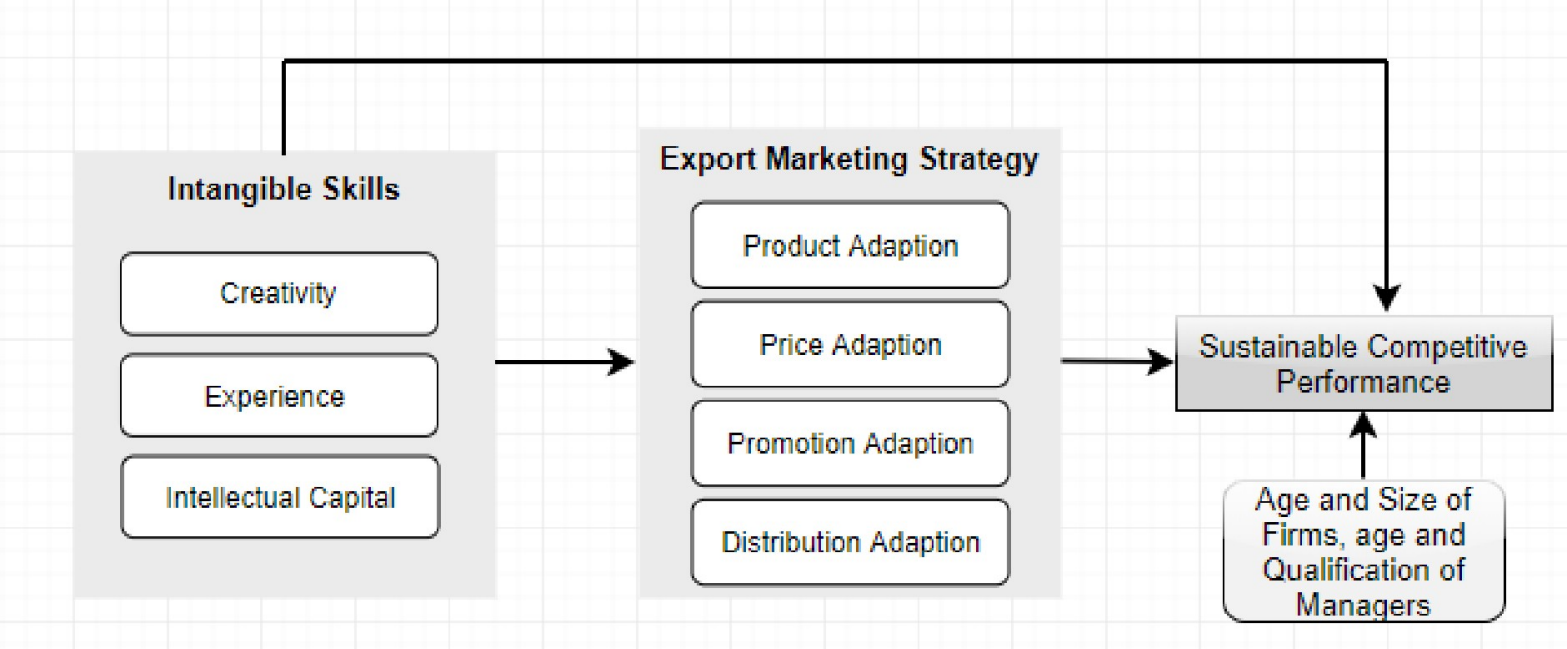
Conceptual framework.

**Fig 3:**
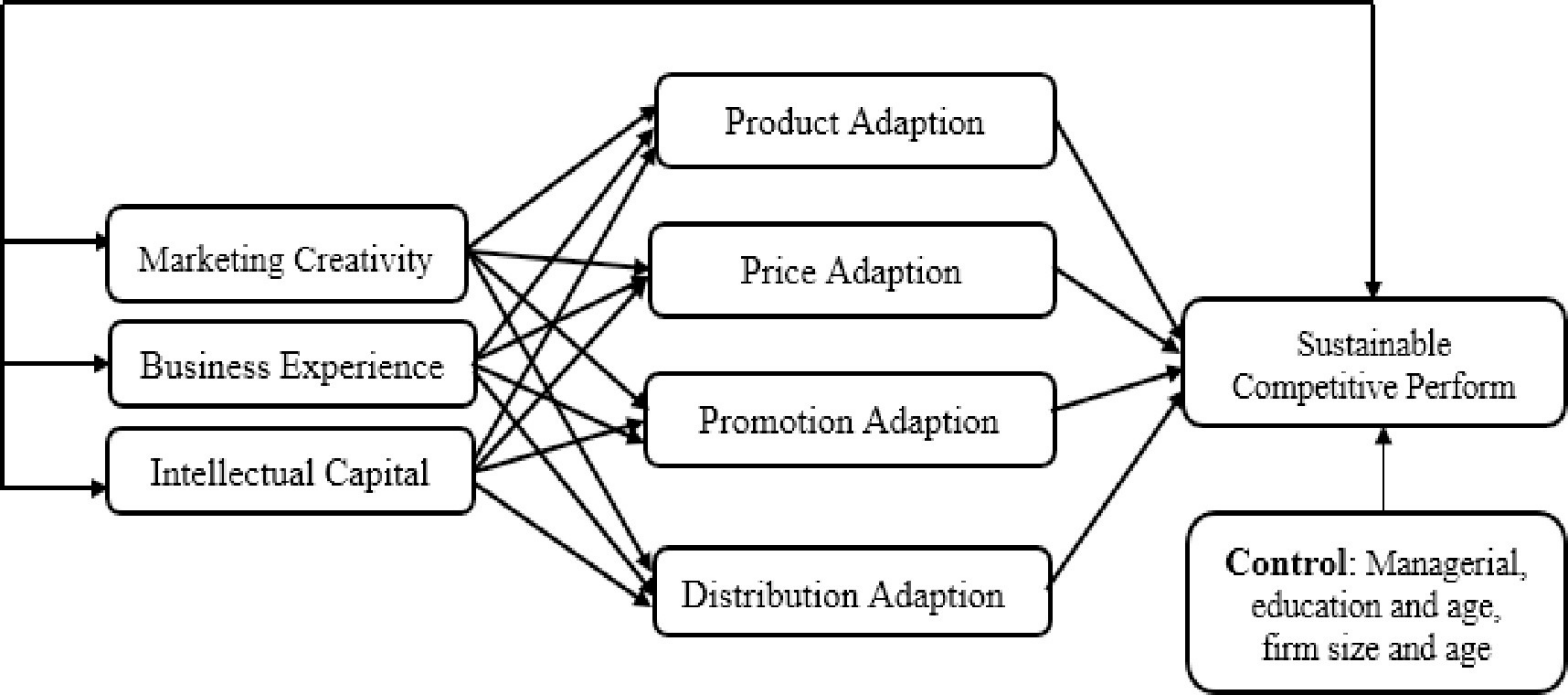
Hypothesized model.

Fig 2 displays conceptual framework of the study where on the left side, independent variables; creativity, experience and intellectual capital, in the middle, export marketing strategy; product, price, promotion and distribution adaption and on the right side, sustainable competitive performance as a dependent variable is shown. Additionally, in Fig 3, we have displayed the hypothesized relationships between the variables. It illustrates how different types of export marketing strategies play the mediating role between managerial skills and sustainable performance in SMEs.

## 3. Methodology

### 3.1 Sample and data

The model of this research is tested on the empirical data gathered from senior marketing managers and financial officers of SMEs. We used a structured questionnaire for data collection with a cover letter stating secrecy of the data and firms information. We followed a convenience sampling approach to collect data for this research. We done pilot testing of the questionnaire from experts in the relevant filed who approved the survey with a few minor suggestions. We distributed hard copies of the questionnaire instead of doing an online survey because of a lower response rate in the SMEs industry [81]. The questionnaire was divided into two sections; in the first section, we have asked age and education of senior managers, nature of industry, size and age of the firms. The second section was related to the main variables; creativity, business experience, intellectual capital and export marketing strategy adaptation. However, to avoid common method variance and to get valid results, we asked marketing managers to fill the part related to creativity, business experience, intellectual capital and export marketing strategy adaptation because of their relevant expertise. However, questions related to sustainable competitive performance were asked financial managers as they are more aware of their financial outcomes and financial reporting. We personally visited the firms and requested responsible managers for filling the survey. We have also asked them that the participation in the survey is volunteer. The questionnaire was prepared in English language because business documents in Pakistan are prepared in English and managers/owners easily understand the language. The questionnaire was checked and approved by the ethical committee of Nankai University for data collection. The committee checked the questionnaire and finalized it for gathering evidence for the conceptualized model. In December, 2019, we distributed 900 questionnaires (300 in each city of which one in each firm) among the enterprises operating in Rawalpindi, Peshawar and Islamabad. Most of senior managers sit in these regions because of the head offices of the enterprises. Due to collectivism culture, Pakistani people are very cooperative and are likely help others. We received 121 questionnaires in the first response (on time of request) from three cities. Due to a busy seclude, many managers said that they will fill the questionnaire and will provide after a week. Hence, after 10 days (first week of January 2020), we visited these firms again, thereby receiving 152 in the three regions, with 30.33% response rate.

In our sample, there were 107 enterprises from manufacturing industry, 94 firms from trading and 92 firms from service industries. There were 32 firms where 20 to 50 employees were working, 44 enterprises have 51 to 100 employees,70 firms were having employees from 101 to 150, 93 enterprises were those where 151 to 200 employees while there were 54 firms where 201 to 250 employees were working. In the data, 65 firms have started their operation since the last 10 years are less, 87 firms aged 11 to 20, 82 were 21 to 30 years old and 59 were those who have started their operation for more than 31 years. Considering the qualification, our sample show that the highest number of managers; 110 have done master and only 14 managers were PhD qualified. Moreover, most of the managers; 118 have 31 to 40 years ages that is the highest number in the sample. Further detail is given in Table 1.

**Table 1:** Frequency analysis.

Particular	Frequency	Distribution
Nature of the industry		
1. Manufacturing	107	36.5
2. Trading	94	32.1
3. Services	92	31.4
Size of the Enterprises		
1. 20–50 employees	32	10.9
2. 51–100 employees	44	15.0
3. 101–150 employees	70	23.9
4. 151–200 employees	93	31.7
5. 201–250 employees	54	18.4
Age of the Enterprises		
1. 10 years and less	65	22.2
2. 11–20 years	87	29.7
3. 21 and 30	82	28.0
4. 31 and above	59	20.1
Qualification of Managers		
1. Bachelor and below	96	32.8
2. Master	110	37.5
3. MS / MPhil	73	24.9
4. PhD	14	4.8
Age of the Managers		
1. 20 to 30 years	54	18.4
2. 31–40 years	118	40.3
3. 41–50 years	78	26.6
4. 51 and above	43	14.7
Total	293	100

### 3.2 Measures

We have used adopted questionnaire/survey in this research and the sources are given below. The questionnaire is freely available and no restriction is applied on using the questionnaire. The original version of the questionnaire was in English and we did not change the language nor any content of the survey.

#### 3.2.1 Creativity

There is not universal definition of creativity in the literature. However, most of the authors are agreed upon the two elements; novelty and usefulness [23, 24, 26]. In the present study, we used three questions (taken from [25] to measure creativity of marketing managers; creative ideas, creative thinking and original ways of doing things).

#### 3.2.2 Business experience

It refers to the knowledge and experience of top managers/owners in international business, trading, export, import, operation, supply, and purchasing and selling. In order to know about experience of the marketing managers, we have asked five questions (adopted from Anwar, Shuangjie [21]) and Ying, Hassan [11]) if they have knowledge or experience of import/export, national and international trade experience etc.

#### 3.2.3 Intellectual capital

as discussed earlier, there are three major dimensions of intellectual capital; human capital, customer capital and structural capital. Intellectual capital is used in large firms with secondary data, while in case of SMEs, due to unavailability of data, self-reported questions are used to measure intellectual capital. We relied on the previous studies [11, 14] where six items were used. However, considering the study, we have slightly modified the items to match it with marketing managers’ characteristics. A sample item is “I have a clear view of what knowledge and competences are the most relevant for the purpose”.

#### 3.2.4 Export marketing strategy adaptation

literature has suggested four major strategies of export marketing; product, price, promotion and distribution that can be considered when a marketer makes new product development or aiming to sale the existing product [2, 82]. In the present study, we also used these strategies (adapted from [3] of which eight items (positioning, design/style, features/characteristics, quality, packaging, brand/branding, labelling, items/models in product line, respectively) are used for product strategy, six items (retail price, wholesale/trade price, profit margins to trade customers, profit margins to end-users, discounts, sales/credit terms, respectively) are used to measure price strategy, five items (advertising, message/theme, sales force structure/management, sales promotion, advertising/promotion budget, respectively) were included for promotion strategy and three items (channels of distribution, type of middlemen and role of middlemen, respectively) we used for distribution strategy.

#### 3.2.5 Sustainable competitive performance

It indicates the outcomes and consequences of a firms in term of profitability, return on investment, return on assets, return on equity and sale growth etc. [11, 81, 83]. In case of listed firms, secondary data makes it easy for researchers to measure performance while in primary data, measuring financial performance is a challenge in research [12, 14]. However, ample evidence are existed that both self-reported and objective measures provide very similar results e.g. [81] Additionally, in case of SMEs, many studies have recommended self-reported measures where firms were asked to rate their performance in terms of return on assets, return on equity, return on investment and sale growth etc. since last three years as compared to the major competitors [12, 14, 21, 77].

### 3.3 Control variables

Controlled variables are used to reduce the chances of spurious results in a data set. Followed the suggestions of previous studies [13, 84], we controlled age and education of the respondents, nature of industry, age and size of the enterprises in our research study. Surprisingly, none of the controlled factors; age and size of SMEs, and age and qualification of managers/owners play a significant role in the model.

## 4. Data analysis

The hypothesized paths have been tested through AMOS.21 that is a specific module of SPSS with deals with SEM analysis.

### 4.1 Descriptive statistics

This tests gave use Mean (M), Standard Deviation (S.D), skewness and kurtosis that are displayed in Table 2. We found that M value of creativity is 3.1938 and its SD is 0.49701. M value of experience is 3.1928 and its SD is 0.39971. M value of intellectual capital is 2.8517 and its SD is 0.37252. The product strategy has the M value 3.1875 and its SD is 0.44297, price has the M value 2.9034 and its SD is 0.37455, promotion has the M value is 3.0538 and its SD is 0.50045 and distribution displays its M value 3.2400 and its SD is 0.48640. Finally, the M value of SCP is 3.5446 and its SD is 0.44856. Additionally, skewness and kurtosis revealed normality of the data because of their values range ±2 that is acceptable as per the suggestion of [85].

**Table 2:** Descriptive statistics.

Variables	Mean	Std. Deviation	Normality		Multicollinearity	
			Skewness	Kurtosis	Tolerance	VIF
Creativity	3.1938	0.49701	-0.441	1.755	0.871	1.148
Experience	3.1928	0.39971	-0.255	1.709	0.881	1.136
Intellectual Capital	2.8517	0.37252	-0.539	3.107	0.946	1.058
Product	3.1875	0.44297	-0.645	1.520	-	-
Price	2.9034	0.37455	0.464	0.522	-	-
Promotion	3.0538	0.50045	0.051	0.231	-	-
Distribution	3.2400	0.48640	-0.551	1.939	-	-
SCP	3.5446	0.44856	0.061	-0.438	-	-

### 4.2 Multicollinearity

It indicates the problem occurred by overlapping between independent variables due to a high correlation [86]. We executed this test in SPSS using regression collinearity option. The results indicate that our data is free of this issue because the tolerance and Variance Inflation Factor (VIF) of all the factors are found in the acceptable range (tolerance values above 0.10 and VIF value below 3) as suggested by Jagpal [86].

### 4.3 Non-response rate

The possibility of this problem is existed in a data set having early responses (first responses) and late responses (after reminder) [87]. To check this issue, we used *T*-test in SPSS by splitting the data into first (121) and late responses (152). We compared the results of the two groups after applying *T*-test but no significant difference is observed in the results which confirmed that the data is free of the non-response bias.

### 4.4 Common method variance

The problem of Common Method Variance (CMV) is existed in a cross sectional data set where a researcher collects data from same respondent, on same time and through a single source [88]. We have collected data from different respondents; one part from marketing manager and other from financial manager, might have no problem of CMV. But for the validity of results, we executed Harman’s single factor test in SPSS to check the theat. We did not find any issue of CMV in the data because the first factor displayed only 20.693% variance (which is below 50%) out of total 8 factors with eigenvalue values above 1.

### 4.5 Confirmatory factor analysis

We executed measurement model in AMOS to assess the factor loading, validity, reliability and model fitness. First we confirmed the fitness of the model in terms of CMININ = 2.245 that is suggested below 3 as per the suggestion of Hu and Bentler [89]. GFI-0.81, AGFI = 0.81, TLI = 0.85, CFI = 0.86 and NFI = .85 provided desirable values as recommended by [90]. RMSEA = .065 and RMR = 0.022 cutoff are below 0.09 [91]. Hu and Bentler [92] and our study met the condition. All the items are statistically significant (p value less than 0.001) that are shown in Table 3.

**Table 3:** Items loadings, validity and reliability.

Variables and Items	Estimate	AVE	√AVE	C.R.
Creativity		0.71	0.84	0.88
c3	0.88*			
c2	0.75*			
c1	0.89*			
Business Experience		0.50	0.71	0.83
be5	0.67*			
be4	0.60*			
be3	0.71*			
be2	0.78*			
be1	0.75*			
Intellectual Capital		0.50	0.71	0.89
ic6	0.66*			
ic5	0.74*			
ic4	0.62*			
ic3	0.85*			
ic2	0.68*			
ic1	0.67*			
Product Adaptation		0.50	0.71	0.89
prd8	0.71*			
prd7	0.80*			
prd6	0.59*			
prd5	0.65*			
prd4	0.68*			
prd3	0.72*			
prd2	0.77*			
prd1	0.70*			
Price Adaptation				
prc6	0.72*	0.50	0.71	0.86
prc5	0.77*			
prc4	0.50*			
prc3	0.88*			
prc2	0.50*			
prc1	0.81*			
Promotion Adaptation		0.53	0.73	0.85
prm5	0.75*			
prm4	0.62*			
prm3	0.91*			
prm2	0.57*			
prm1	0.74*			
Distribution Adaptation		0.65	0.81	0.84
dst3	0.88*			
dst2	0.56*			
dst1	0.93*			
Sustainable Competitive Performance		0.52	0.72	0.86
scp1	0.79*			
scp2	0.81*			
scp3	0.85*			
scp4	0.51*			
scp5	0.70*			
scp6	0.60*			

* = significant at p value 0.001, AVE = Average variance extracted, CR = Composite reliability.

Validity and reliability of the model are shown in Table 3. Convergent Validity: it indicates the average variance explained by observed variables (items) in an unobserved variable (construct). If the items explain average variance of 0.50 or above, it means they explain sufficient variance e.g. Average Variance Extracted (AVE) in the unobserved variable [93]. Our data met this condition as all the constructs provided satisfactory values.

Discriminant Validity: it indicates if the observed variables explain unique variance in their respective constructs rather than overlapping with each other. The threshold value of discriminant validity is 0.70 or above [93]. Our data also met this criterion as all the constructs have satisfactory values.

Discriminant Validity: it illustrates internal consistency among the unobserved variables that are loaded on a particular construct. A value above 0.70 displays adequate reliability [94] and our study met the condition.

### 4.6 Correlations

We executed correlations between the variables; creativity, experience, export marketing strategy (product, price, promotion and distribution) and sustainable competitive performance using SPSS (see Table 4). Our results specify that creativity is significantly related to the product strategy (r = 0.404, p <0.05) and distribution (r = 0.189, p <0.05) but insignificantly related to price (r = 0.047, p >0.05) and promotion (r = 0.077, p >0.05). Experience is significantly related to all the dimensions of export marketing strategy; product (r = 0.369, p <0.05), price (r = 0.281, p <0.05), promotion (r = 0.238, p <0.05) and distribution (r = 0.218, p <0.05). Intellectual capital is only significantly related with product (r = 0.259, p <0.05) while insignificantly related to other dimensions of export marketing strategy; price (r = 0.037, p >0.05), promotion (r = 0.088, p >0.05) and distribution (r = 0.096, p >0.05). All the intangible skills of marketing managers; creativity, experience and intellectual capital are significantly related to SCP (r = 0.226, p <0.05, r = 0.315, p <0.05 & r = 0.177, p <0.05) respectively. The correlations values confirm the absence of multicollinearity problem because none of the values is greater than 0.80.

**Table 4:** Correlations.

Variables	1	2	3	4	5	6	7	8	9	10	11	12
1. Size	1											
2. AgeF	0.161**	1										
3. Education	0.158**	0.058	1									
4. AgeR	0.102	0.111	0.363**	1								
5. Creativity	0.075	0.018	0.001	0.089	1							
6. Experience	0.059	-0.005	0.044	0.044	0.327**	1						
7. IntCapital	-0.096	-0.116*	0.062	-0.032	0.203**	0.175**	1					
8. Product	-0.066	-0.043	0.066	0.001	0.404**	0.369**	0.259**	1				
9. Price	0.152**	0.041	0.050	0.019	0.047	0.281**	0.037	0.177**	1			
10. Promt	0.080	0.099	0.035	0.043	0.077	0.238**	0.088	0.107	0.162**	1		
11. Distrib	0.000	0.026	-0.074	0.019	0.289**	0.218**	0.096	0.387**	0.235**	0.265**	1	
12. SCP	0.073	0.020	0.009	0.040	0.226**	0.315**	0.177**	0.594**	0.329**	0.277**	0.403**	1

Note: ** Correlation is significant at the 0.01 level (2-tailed).

* Correlation is significant at the 0.05 level (2 tailed). AgeF = Age of firms, AgeR = Age of the respondents.

### 4.7 Structural model

**Fig 4:**
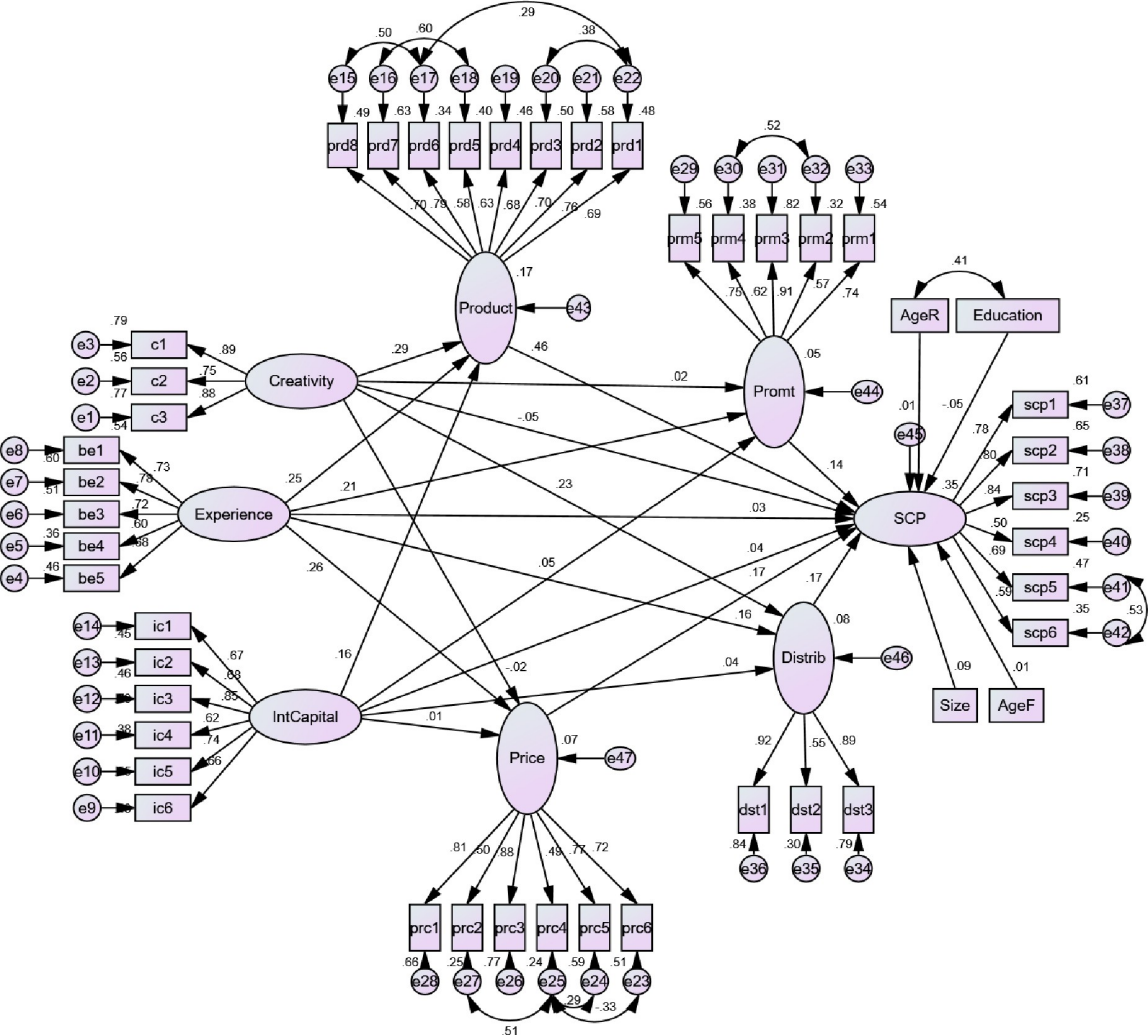
Structural model.

For testing the hypothesized associations, we applied structural model in AMOS that is shown in Fig 4. However, we first confirmed the fitness of the model in terms of CMIN = 2.135 that displayed an acceptable value (below 3), GFI = 0.82, AGFI = 0.80, CFI = 0.85, TLI = 0.84 and NFI = 0.83 which revealed desirable value (above 0.90) and RMR = 0.35 and RMSEA = .062 also gave use desirable value (below 0.09) as per the suggestion of Bollen and Stine [91] and Hu and Bentler [92].

Our research revealed (see Table 5) that none of the variables; creativity, experience and intellectual capital has a significant direct influence on SCP (β = -0.046, p > 0.05, β = 0.027, p > 0.05 and β = 0.041, p > 0.05) and thus rejected the hypotheses 1, 2 and 3 respectively.

**Table 5:** Hypotheses results.

Paths	Direct	indirect	Total
SCP ← Creativity	-0.046	0.175**	0.129*
SCP ← Experience	0.027	0.214**	0.242**
SCP ← Intellectual Capital	0.041	0.087	0.128
Product ← Creativity	0.294**	-	0.294**
Price ← Creativity	-0.016	-	-0.016
Promotion ← Creativity	0.015	-	0.015
Distribution ← Creativity	0.231*	-	0.231*
Product ← Experience	0.247**	-	0.247**
Price ← Experience	0.264**	-	0.264**
Promotion ← Experience	0.210*	-	0.210*
Distribution ← Experience	0.160	-	0.160
Product ← Intellectual capital	0.158*	-	0.158*
Price ← Intellectual capital	0.005	-	0.005
Promotion ← Intellectual capital	0.051	-	0.051
Distribution ← Intellectual capital	0.036	-	0.036
SCP ← Product	0.461**	-	0.461**
SCP ← Price	0.167*	-	0.167*
SCP ← Promotion	0.140	-	0.140
SCP ← Distribution		-	0.171*
SCP ← Age of firms		-	0.011
SCP ← Size of firms	0.171*	-	0.093
SCP ← Qualification of Managers	0.011	-	-0.046
SCP ← Age of Managers	0.014	-	0.014

** p value (0.001)

* p value (<0.05).

The impact of creativity on product strategy is significant (β = -0.046, p < 0.05), insignificant on price (β = -0.16, p > 0.05), also insignificant on promotion (β = 0.015, p > 0.05) while significant on distribution (β = 0.231, p < 0.05) which partially supported H4.

The impact of experience on product strategy is significant (β = 0.247, p < 0.05), significant on price (β = 0.264, p < 0.05), also significant on promotion (β = 0.210, p < 0.05) while insignificant on distribution (β = 0.160, p > 0.05) which also partially supported H5.

The impact of intellectual capital on product strategy is significant (β = 0.158, p < 0.05), insignificant on price (β = 0.005, p < 0.05), also insignificant on promotion (β = 0.051, p > 0.05) as well as also insignificant on distribution (β = 0.036, p > 0.05) which partially supported H6.

The three dimensions of export marketing strategy have a significant influence on SCP; product (β = 0.461, p < 0.05), price (β = 0.167, p < 0.05), distribution (β = 0.171, p < 0.05), while promotion has not a significant impact on SCP (β = 0.140, p > 0.05), hereby partially supporting H7.

Considering the mediating role of export marketing strategy, our results reveal that the indirect influence of creativity on SCP through the export marketing strategy (product, price, promotion and distribution) is significant (β = 0.175, p < 0.05) while the direct impact of creativity on SCP remained insignificant, resulting a full mediation, and supported H8. Similarly, the indirect influence of experience on SCP through the export marketing strategy is also significant (β = 0.214, p < 0.05) and the direct influence remained insignificant, resulting a full mediating and supported H9. However, our research reveals that intellectual capital has not a significant indirect influence on SCP nor displays a direct significant role. Hence, we scrutinize that the export marketing strategy does not mediate the path between intellectual capital and SCP which rejected H10. Surprisingly, all the controlled factors; age and age of the enterprises and qualification and age of the managers show insignificant role in the model. The intangible resources; creativity, experience and intellectual capital explain 35% variance in SCP through the export marketing strategy and in the presence of the controlled variables. See Table 6 for hypotheses remarks.

**Table 6:** Hypotheses remarks.

Hypotheses	Remarks
H1. Creativity significantly contributes to the sustainable competitive performance.	NS
H2. Business experience significantly contributes to the sustainable competitive performance.	NS
H3. Intellectual Capital significantly contributes to the sustainable competitive performance.	NS
H4. Creativity significantly facilitates marketing managers in export marketing strategy adaption (product, price, promotion and distribution)	PS
H5. Business experience significantly facilitates marketing managers in export marketing strategy adaption (product, price, promotion and distribution).	PS
H6. Intellectual Capital significantly facilitates marketing managers in export marketing strategy adaption (product, price, promotion and distribution).	PS
H7. Export marketing strategy adaption (product, price, promotion and distribution) has a significant influence on sustainable competitive performance.	PS
H8. Export marketing strategy adaption mediates the relationship between creativity and sustainable competitive performance.	S
H9. Export marketing strategy adaption mediates the relationship between experience and sustainable competitive performance.	S
H10. Export marketing strategy adaption mediates the relationship between intellectual capital and sustainable competitive performance.	NS

Note: NS = Not supported, PS = Partially supported, S = Supported.

### 4.8 Robustness checks

To enhance validity of the findings, we executed regression analysis via SPSS and examined the mediating role of each export marketing strategy; product adaptation, promotion adaptation, price adaptation and distribution adaptation between each intangible skill; creativity, experience and intangible capital and sustainable business performance. We executed separate models for each export strategy in the presence of the controlled factors; age and educational background of the respondents, age and size of the enterprises. The results of these tests are discussed in Table 7.

**Table 7:** Regression analysis.

	Model 1	R^2^	RΔ	Model 2	R^2^	RΔ	Model 3	R^2^	RΔ	Model 4	R^2^	RΔ
Step 1	3.427***	0.007	0.007	3.427	0.007	0.007	3.427			3.427		
AgeR	0.018			0.018			0.018			0.018		
Education	-0.008			-0.008			-0.008			-0.008		
AgeF	0.002			0.002			0.002			0.002		
Size	0.026			0.026			0.026			0.026		
Step 2	1.807***	0.132***	0.125***	1.807	0.132***	0.125***	1.807	0.132***	0.125***	1.807	0.132***	0.125***
AgeR	0.012			0.012			0.012			0.012		
Education	-0.015			-0.015			-0.015			-0.015		
AgeF	0.010			0.010			0.010			0.010		
Size	0.022			0.022			0.022			0.022		
Creativity	0.100			0.100			0.100			0.100		
Experience	0.285***			0.285***			.285***			0.285***		
IntCapital	0.145**			0.145**			0.145**			0.145**		
Step 3	1.170***	0.384***	0.252***	1.176	0.195***	0.064***	1.490	0.171	0.039***	1.216	0.233***	0.102***
AgeR	0.026			0.013			0.010			0.008		
Education	-0.039			-0.017			-0.015			0.000		
AgeF	0.014***			0.007			0.001			0.005		
Size	0.042			0.009			0.017			0.024		
Creativity	-0.062			0.115			0.104			0.028		
Experience	0.121**			.198***			0.232***			0.232***		
IntCapital	0.050			0.141**			0.128			0.132**		
Product	0.593***			--			-			-		
Price	-			0.318***			-			-		
Promotion	-			-			0.184***			-		
Distribution	-			-			-			0.311***		

Note: Dependent variables in Model 1 = product adaptation, Model 2 = Price adaptation, Model 3 = Promotion adaptation, Model 4 = Distribution adaptation.

*** p value (0.001)

** p value (<0.05).

The results of the regression analysis displayed a slightly different but overall, we found a good linkage between regression and the structural model. Considering the first model where the mediating role of product adaptation has been assessed, our findings show that product adaptation is partially mediated the relationship between intellectual capital and SCP while fully mediates the link between intellectual capital and SCP. In the second model, we found that price adaptation strategy plays a partial mediating role between experience and intellectual and SCP while it fully mediates the link between creativity and SCP. In the third model, our findings display that promotion strategy is a partial mediator between experience and SCP while it plays a full mediating role between creativity, intellectual capital and SCP. Finally, in the fourth model, we found that distribution strategy is a partial mediator between experience and intellectual capital and SCP while it is a partial mediator between creativity and SCP. None of the controlled factors displayed a significant role in the model.

## 5. Discussion and concluding remarks

Steered by the RBV theory, the present study unleashed the importance of intangible resources; creativity, business experience and intellectual capital in sustainable competitive performance through export marketing strategy adaption. Previous studies have contributed to the RBV theory through empirical and theoretical studies, both in emerging and advanced economies [11, 13, 71, 84]. However, in particular, how the intangible skills; creativity, business experience and intellectual capital through export marketing strategy adaptation influence sustainable competitive performance has been missed from the RBV literature. Therefore, testing the unexploited and neglected zone, the present study contributes to the RBV theory in an empirical way—using data of Pakistani SMEs. It advances the understanding of the theory in the literature of emerging SMEs and export marketing strategy adaptation. Our research reveals that the particular intangible resources are might be a part of the theory that can facilitate enterprises in adaptation of export marketing strategy in emerging markets.

Our research scrutinized that creativity has not a significant direct influence on SCP in SMEs which rejects the first hypothesis. The findings do not favor Castillo-Vergara, Alvarez-Marin [95] who described that creativity is the key to profitability in business industry. However, our findings are matched with several similar studied. For instance, Khedhaouria, Gurău [96] conducted a study in French SMEs and scrutinized that creativity does not directly influence firm performance but entrepreneurial orientation fully mediates the path. Boso, Donbesuur [47] revealed that organizational creativity first configures new product development speed which in turn influences new product performance. The nation was also supported by Cheng and Yang [97] who concluded that new product development activities and speed mediate the connection between creative process and new product performance. In line with Dul and Ceylan [98] who demonstrated that creativity spurs novelty of managers related to products and services, resulting in a sustainable positon of the products in the markets. Our research also displays that marketing managers with creativity do not directly affect firm performance but indirectly through export marketing strategy adaption such as product, process, price and distribution.

Our research also revealed that experience does not directly influences sustainable competitive performance but indirectly influences thorough the adaption of export marketing strategy. Our findings do not relate Dhliwayo [99] who stated that business experience has a significant influence on financial performance. Our research reveals that experience has an indirect influence on sustainable performance in business industry where the adaption of export marketing strategy plays a full mediating role. However, [21] demonstrated that new opportunity recognition partially mediates the link between managerial experience and sustainable performance. Matching Feurer, Schuhmacher [58] who described that adequate knowledge and experience are very crucial in building innovative products and marketing strategy. It empowers managers in producing tolerably match products for customers and markets that result superior performance.

Surprisingly, our research indicated that intellectual capital does not influence SCP nor has influence on the adaption of export marketing strategy. Our results do not support the previous studies where a positive and significant relationship between intellectual capital and performance has been reported [14, 22, 65, 67]. Our findings are different from Rexhepi, Bexheti [67] who have stated that IC is a crucial predictor of global marketing strategy and influences new products activities that results high performance. Our findings do not favor Gomezelj Omerzel and Smolčić Jurdana [60] who revealed that innovativeness and new initiatives of organizations are depended on their intellectual capital. We resulted that neither IC is a significant predicator of performance neither the adaption of export marketing strategy mediates the path. Our findings are different than Tsai and Hsu [63] who scrutinized that marketing managers use intellectual skills for bringing innovation and building new products that results high growth. Marketing managers in Pakistan do not get benefits of intellectual capital during the adaption of export marketing strategy. However, they rely on their experience and creativity when they intend to adapt export marketing strategy to benefits their enterprises. SMEs can investigate the reasons behind lacking importance of intellectual capital in the adaption of export marketing strategy and performance. Because the adaption of export marketing strategy enables enterprises to gain a sustainable competitive positon. This claim is supported by several research studies such as Boso, Adeola [4] and Zou, Fang [74] export marketing strategy is the key to financial performance sustainable positon in a competitive market.

### 5.1 Implications for practice

The results of this study have some practical implications for the firms’ policies and managers. First, our findings show that creativity has not a significant positive influence on SCP in SMEs. SMEs are required to carefully investigate the new ideas and activities of their marketing managers, and ensure if these ideas and new initiative do not properly match customer exceptions and markets demands. Though creativity indirectly significantly contributes to SCP through the export marketing strategy especially distribution. Second, our study unleashed that experience also does not directly contribute to SCP but indirectly through the adaptation of export marketing strategy. Therefore, SMEs need to hire experience marketing managers who can build and formulate effective export strategies in order to spur the profitability. Third, our findings show that intellectual capital does not facilitate marketing managers in increasing SCP directly or indirectly through export marketing strategy adaptation. It posits that intellectual capabilities of marketing managers are not necessarily mean high performance nor useful for export marketing strategy. However, it does not mean SMEs should regret intellectual skills of marketing managers as it can enable SMEs in creating unique product, as revealed by this research. Overall, our results display that the intangible skills of marketing managers; creativity and experience indirectly contributes to SCP through the adaptation of export marketing strategy while intellectual capital does not show any significant role, except important for building product. To summarize, our results suggest the following key implications for practice;

- SMEs need to investigate and check why new and creative ideas of marketing managers’ do not work effectively. Especially cost vs benefits analysis of the creative ideas can be done to explore the reasons of negative insignificant connection between creativity and SCP.
- SMEs are recommended to hire experienced and creative marketing managers they have capabilities to adopt export marketing strategy that results in sustainable performance and superior profitability.
- In particular, where product adaptation strategy (e.g., building new or modifying an existing one) is a key goal, SMEs need to emphasize on creative, experienced and intellectual marketing managers.
- However, when SMEs formulate their pricing strategy and intend to promote a new or existing product, they are strongly advised to focus on experienced marketing managers.
- For distribution strategy, our findings recommend creative marketing managers as compared to intellectual and experienced one.
- Ultimately, our research recommends the adaptation of export marketing strategy because it significantly facilitates enterprises in achieving superior performance.
- SMEs do not to be curious with demographic factors of marketing managers such as age and education because these factors are not significantly reported in our results.
- Moreover, our research gives alarming signals to marketing managers to get ready for CPEC—a project aiming growth of international trade between China and Pakistan.

### 5.2 Limitations and direction for future studies

This research suggested praiseworthy implications for practicing after evaluating the role of influence the intangible skills; creativity, business experience and intellectual capital on sustainable competitive performance through export marketing strategy adaptation. Despite the meaningful implications, this research is not free of constraints that need to be addressed in additional studies. The first limitations of this research comes from testing the particular intangible skills such as creativity, business experience and intellectual capital. Other intangible skills such as entrepreneurial orientation and network ties can be considered in additional studies as these factors significantly facilitate newness and sustainable performance of SMEs in emerging markets [100, 101] can be considered for the adaptation of export marketing strategy. Moreover, if possible and have not a critical issue of resource constraints, tangible resources such as financial availability and technology can be considered because of their significant role in organizational success and new products and new process [102]. Parsimoniously, relative importance and consequences of both tangible and intangible resources can be evaluated in export marketing strategy adaptation and competitive performance. The second limitations of this research comes from the target population as we surveyed only marketing managers of Pakistani SMEs and despite potential implications for Chinese firms, we could not interview them. We suggest data collection of marketing managers of Chinese firms to articulate the results in a better way. Because both the enterprises are planning to participate in CPEC and improve their sale performance. Additionally, other actions and perceived factors can be considered in the model to articulate the insights and consequences in a better approach. Perhaps demographic and behaviors that can influence the adaptation of export marketing strategy and business performance.

## 6. Conclusion

The aim this research was to test the influence of the intangible skills; creativity, business experience and intellectual capital on sustainable performance with a mediating role of export marketing strategy adaptation. We tested the model on the theme of RBV theory by collecting empirical evidence from SMEs operating in the emerging market Pakistan. To test the hypothesized relationship, we used structural equation model in AMOS. The results show that none of the intangible skills; creativity, business experience and intellectual capital directly influence sustainable performance of SMEs. Creativity significantly influences product and distribution strategy, business experience significantly influence product, price and promotion strategy and intellectual capital significantly influences only product strategy. Export marketing strategy significantly improves sustainable performance of SMEs. Moreover, export marketing strategy fully mediates the relationship between creativity, experience and sustainable performance while intellectual capital does not mediate the paths. Our research recommends SMEs to focus on marketing managers with high intangible skills and especially creativity, experience and intellectual capital should be considered.
